# The Vitiligo in Senegal

**DOI:** 10.5402/2012/932163

**Published:** 2012-01-11

**Authors:** S. O. Niang, Maodo Ndiaye, Fatimata Ly, Moussa Diallo, Sonia Bouksani, Assane Diop, Boubacar Ahy Diatta, Mame Thierno Dieng, Assane Kane

**Affiliations:** ^1^Department of Dermatology, Aristide Le Dantec Hospital, BP 3001, Dakar, Senegal; ^2^Department of Dermatology, Institut d'Hygiène Sociale, Dakar, Senegal

## Abstract

The aim of our study was to determine the epidemiological and clinical aspects of vitiligo in the largest dermatology department of Senegal. A cross-sectional and descriptive study in a period of 5 months was performed covering all the vitiligo cases. Fifty patients were identified (26 women and 24 men). The mean age was 26.5 years. A family history of vitiligo was found in 11 cases and a psychoaffective disturbance in 6 cases. The clinical forms distinguished were generalized vitiligo (*n* = 33), localized vitiligo (*n* = 16), vitiligo universalis (*n* = 4), and segmental vitiligo (*n* = 1). The Koebner phenomenon was found in 7 cases. Associated diseases were atopic dermatitis (*n* = 2), contact dermatitis (*n* = 1), diabetes (*n* = 1), and Graves' disease (*n* = 1). The disgraceful character of Vitiligo was the predominance of generalized forms and the elective localization in sun-exposed areas. The family character, the psychoaffective disturbances, the Koebner phenomenon increased by the lifestyle and the itching dermatosis were the aggravating factors.

## 1. Introduction

Vitiligo is a depigmenting condition characterized by a loss of functional melanocytes or by the decrease of melanin in the epidermis. Its prevalence is about 0.1 to 2% depending on the country [1]. Although asymptomatic and non-disabling, vitiligo is a very debilitating disease because of its exhibited and highly unpleasant character on dark skin. Nowadays, no pathophysiological mechanism has been yet elucidated. Antibodies against melanocytes and free radicals affecting cell membranes or cytoplasmic components have been suspected till today [2–4]. There is no efficient treatment. Due to its benignity, very few studies have been devoted to this disease in Africa and none in Senegal. The aim of our study was to report the epidemiological and clinical aspects of vitiligo in Senegal. 

## 2. Patients and Methods

We conducted a cross-sectional and descriptive study, identifying all the cases of vitiligo seen over a period of five months in the dermatology department of the university teaching hospital Le Dantec. It is the largest referral center of dermatology in Dakar, the capital of Senegal. The diagnosis of vitiligo was based on clinical and histological data. The examination searched for the family history of vitiligo, a psychoaffective shock in the 3 years preceding the onset of lesions and a Koebner phenomenon. It also searched for another associated autoimmune disease such as type I diabetes, Graves' disease, and autoimmune thyroiditis. 

## 3. Results

**Figure 1:**
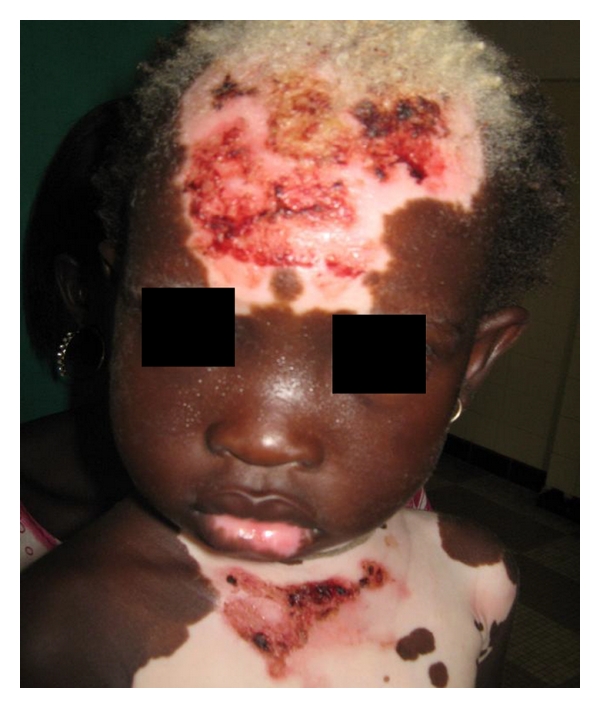
Generalized vitiligo of a two years old children (a family history vitiligo).

**Figure 2:**
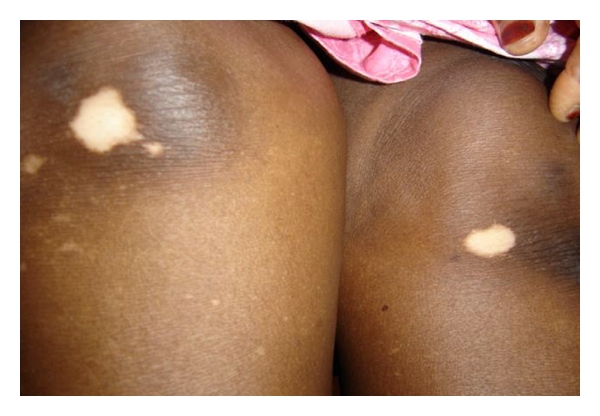
Localized vitiligo.

Out of 4192 patients seen during this period, 50 presented a vitiligo corresponding to a frequency of 1.2% among them 26 women (52%) and 24 men with a sex ratio of 0.9. The mean age of the lesion's inception was 26.5 years, ranging from 15 days to 66 years. Patients under 20 years were 21 (42%). A family history of vitiligo (Figure 1) was noted in 11 cases (22%). Among them, one was a homozygote twin. A psychoaffective disturbance was noted in the patients aged over 18 years old in 6/37 cases (16.2%) in the 3 years preceding the onset of the lesions. It was mourning in 3 cases, a job loss, a pregnancy, or a change of residence each in 1 case. The clinical presentation was a generalized vitiligo affecting more than one region of the body in 33 cases (66%), a localized vitiligo (Figure 2) in 16 cases (32%), a vitiligo universalis in 4 cases (8%) and a segmental vitiligo localized in a dermatome in 1 case (2%) (Table 1). The Koebner phenomenon was found in 7 cases (14%). The distribution of the cases according to the topography is illustrated in Table 2. Other associated troubles were type I diabetes in 1 case, contact dermatitis in 3 cases, lichen planus in 2 cases, maniac depressive disorder in 1 case. 

## 4. Discussion

We report 50 cases of vitiligo observed over a period of 5 months. The characteristics of our study are the predominance of the generalized form, the rarity of the segmental form, the elective topography in sun-exposed areas, the existence of a Koebner phenomenon, and an anterior psychoaffective shock in about a quarter of cases each. Our study was limited by its short duration which did not allow us to evaluate the lesions' outcome on the long run. The 1.2% frequency is close to the one registered by Sehgal et al. in India [5] and the frequency of 1 to 2% reported worldwide [6]. The female predominance was also observed by Kar [7] who noted 51.6% of women against 48.35% of men. Boisseau-Garsaud et al. [8] then explained this difference by a greater concern for aesthetics in women who consult more. The onset mean of our patients was 26.5 years, close to the one of 29 years noted by Boisseau-Garsaud et al. [8]. This distribution is consistent with the usual data that locates the onset age of vitiligo between 10 and 30 years. The congenital vitiligo observed in our study is unusual in the western series [6]. Its frequency may be underestimated due to the difficulty of identification on the Caucasian newborn skin. 

The existence in our study of vitiligo in another family member (22%) and even in a homozygous twin highlights the genetic predisposition of this disease. Besides, Mosher and Jaigirdar have observed it, respectively, in 21% and 24% [6, 9]. Indeed, a mutation in the gene *NAPL1* (Nacht leucine-rich-repeat protein 1) has been identified as correlated with the risk of developing vitiligo [10]. The concept of sustainable stress noted in the past medical history is present in all the patients of Papadopoulos's series [11, 12] who found economic difficulties in 35% of cases, mourning in 42.5% of cases, a difficult pregnancy in 16% cases and a change of residence in 26% of cases. Koebner's phenomenon was objectified in the studies of Gauthier [13] and Jaigirdar et al. [9] in 30% and 100% of cases. Maria et al. [14] noted in her comparative series a Koebner phenomenon in 66.2% of the bilateral vitiligo and 44.4% in the unilateral type of vitiligo. In itching dermatosis such as eczema and lichen planus, the scratching lesions act by Koebner's phenomenon. Some daily gestures such as friction with the bath glove in African can be aggravating factors. The rate of different clinical forms varies depending on the series. Our results are superposed to those of Martis [15] which found 65% of generalized forms, 32% of localized forms, and 3% of segmental forms. They are different from those of Handa and Kaur [16] who found in his series of 1436 cases diffused lesions in 69.8% and only 14.9% of localized forms [11, 15]. The elective topography in sun-exposed areas was also observed by Kar [17]. According to some authors, the sun would act by a Koebner phenomenon [6, 15]. The palmoplantar localizations and mucous membranes (vulva, lips) seem more frequent in patients with racial pigmentation [6, 15]. Nevertheless, labial involvement may be overestimated because of the frequent confusion with other postinflammatory cheilitis. The association with other autoimmune diseases reported elsewhere was infrequent in our study. Only one case of type II diabetes and one case of Graves' disease were identified in our series. 

## 5. Conclusion

The displaying character of vitiligo in our study evidenced by the predominance of generalized forms, elective topography in the sun-exposed areas, and the majority involvement in women focuses more on aesthetic prejudice induced on dark skin. 

The development of better treatment protocols is necessary. 

## Figures and Tables

**Table 1:** Different clinical forms of vitiligo.

Clinical forms	Patients	%
Generalized vitiligo	33	66%
Localised vitiligo	16	32%
Vitiligo universalis	4	8 %
Segmental vitiligo	1	2%

**Table 2:** Distribution lesions of vitiligo.

Localization of lesions	Patients	%
Sun-exposed areas	37	61%
Limbs	16	26%
Trunk	14	23%
Genital area region	11	18%
Hair	15	25%
